# An isolated perfused pig heart model for the development, validation and translation of novel cardiovascular magnetic resonance techniques

**DOI:** 10.1186/1532-429X-12-53

**Published:** 2010-09-17

**Authors:** Andreas Schuster, Inga Grünwald, Amedeo Chiribiri, Richard Southworth, Masaki Ishida, Gunnar Hay, Nicole Neumann, Geraint Morton, Divaka Perera, Tobias Schaeffter, Eike Nagel

**Affiliations:** 1 King's College London BHF Centre of Excellence, NIHR Biomedical Research Centre and Wellcome Trust and EPSRC Medical Engineering Centre at Guy's and St. Thomas' NHS Foundation Trust, Division of Imaging Sciences, The Rayne Institute, London, UK; 2 German Heart Institute, Berlin, Germany; 3 King's College London BHF Centre of Excellence, NIHR Biomedical Research Centre and Department of Cardiology, Guy's and St. Thomas' NHS Foundation Trust, London, UK

## Abstract

**Background:**

Novel cardiovascular magnetic resonance (CMR) techniques and imaging biomarkers are often validated in small animal models or empirically in patients. Direct translation of small animal CMR protocols to humans is rarely possible, while validation in humans is often difficult, slow and occasionally not possible due to ethical considerations. The aim of this study is to overcome these limitations by introducing an MR-compatible, free beating, blood-perfused, isolated pig heart model for the development of novel CMR methodology.

**Methods:**

6 hearts were perfused outside of the MR environment to establish preparation stability. Coronary perfusion pressure (CPP), coronary blood flow (CBF), left ventricular pressure (LVP), arterial blood gas and electrolyte composition were monitored over 4 hours. Further hearts were perfused within 3T (n = 3) and 1.5T (n = 3) clinical MR scanners, and characterised using functional (CINE), perfusion and late gadolinium enhancement (LGE) imaging. Perfusion imaging was performed globally and selectively for the right (RCA) and left coronary artery (LCA). In one heart the RCA perfusion territory was determined and compared to infarct size after coronary occlusion.

**Results:**

All physiological parameters measured remained stable and within normal ranges. The model proved amenable to CMR at both field strengths using typical clinical acquisitions. There was good agreement between the RCA perfusion territory measured by selective first pass perfusion and LGE after coronary occlusion (37% versus 36% of the LV respectively).

**Conclusions:**

This flexible model allows imaging of cardiac function in a controllable, beating, human-sized heart using clinical MR systems. It should aid further development, validation and clinical translation of novel CMR methodologies, and imaging sequences.

## Background

Coronary artery disease (CAD) with subsequent myocardial infarction and heart failure constitutes a leading cause of death in the western world [1]. Myocardial ischaemia is the major pathophysiological mechanism underlying these cardiovascular diseases.

Cardiovascular magnetic resonance (CMR) is being used increasingly in the diagnosis and assessment of CAD as it has the unique capability of assessing myocardial function, viability and perfusion in one session [2]. It also compares favourably with other non-invasive methods [3-6]. This high-resolution technique has evolved rapidly over the past few years with the development of new hardware, contrast agents, acquisition sequences and new post-processing tools. These recent and ongoing advances bring the promise of improved diagnostic accuracy and understanding of the pathophysiology and, most importantly, improved management of the disease [7]. However these new methods have to be developed and validated before finally being translated to patients.

Novel CMR techniques and imaging biomarkers are often validated in small animal models or empirically in patients. Direct translation of small animal CMR imaging protocols to humans is rarely possible. On the other hand, validation of novel imaging techniques in humans, for example quantitative perfusion, novel sequences, optimized contrast agent injection schemes or responses to alterations of blood flow requires large patient populations and is occasionally not possible for ethical reasons.

In order to observe cardiovascular physiology and pathophysiology in a controlled fashion, complex experimental models are necessary to mimic the human situation as closely as possible. The porcine heart closely resembles the human heart from the point of view of size, physiology and anatomy and consequently pigs are frequently used for in vivo cardiovascular research [8].

Oscar Langendorff introduced a model of retrograde perfusion of mammalian hearts via the Aorta with Krebs Henseleit solution in 1895 [9]. This preparation was refined by using whole blood as a perfusate and by either perfusing the coronary arteries directly or filling of the atria and chambers in a working heart mode in pigs [10-12].

Explanted haemoperfused pig heart models have been developed to represent *in situ *physiological cardiac function *ex vivo *[10]. They have been used for various purposes including studies of cardiac physiology and comparison of different techniques of donor heart preservation [11], but have not yet been exploited for clinical CMR, most likely due to practical difficulties such as perfusion rig design within the magnetic resonance (MR) environment.

The purpose of this work is to design, build and test the feasibility of a novel MR compatible explanted, beating pig heart model that allows control of regional blood flow, oxygenation and nutrient delivery. Such a model would allow validation of CMR derived parameters against gold standards and easy translation of novel CMR methods to patients, using equipment and imaging sequences that mimic routine clinical practice.

## Methods

The work was carried out in two phases.

In *phase 1 *(n = 6 hearts) we validated the model against several in-vivo physiological parameters, and characterised its stability outside of the MR environment. In *phase 2 *(n = 6 hearts) after re-developing the model to be MR compatible we determined its feasibility at 3 Tesla (n = 3 hearts) and 1.5 Tesla (n = 3 hearts) clinical MR scanners.

Experiments were conducted after approval by the relevant authorities. All experiments were performed in compliance with the World Medical Association Declaration of Helsinki regarding ethical conduct of research involving animals.

### Phase 1

#### Heart removal and preparation

Hearts were harvested from Large White Cross Landrace pigs weighing between 40 and 60 kg (average weight of 51 ± 6 kg) as previously described [12]. In brief, pigs were premedicated with 6 mg/kg azaperone and 0.05 mg/kg atropine, and then anaesthetised with 10 mg/kg ketamine and ventilated with N_2_O at 4.5 l/min and O_2 _at 4 l/min. General anaesthesia was maintained with 0.8-1.1 vol.% isoflurane and 1-3 μg/kg/h fentanyl. The animals were heparinised with 5,000 IU of heparin, the thorax was opened and 1.8 l of autologous blood was collected via the superior vena cava into 1l plastic bottles containing 10,000 I.U. heparin. The pericardium was opened and the heart was removed after transection of the great heart vessels.

The heart was immediately immersed in iced 0.9% saline solution and the coronary arteries were perfused with cold (4°C) Custudiol solution (HTK solution, Dr. Franz Köhler Chemie GmbH, Alsbach-Hähnlein, Germany).

Hearts were transported to the laboratory and prepared for perfusion as follows. The pulmonary artery was shortened to 3-3.5 cm and the aorta to approximately 4 cm. Both vessels were attached to an appropriate cannula. The remaining orifices were sewn closed.

Warm ischaemic time from excision to beginning of cardioplegic infusion was less than one minute. Cold ischaemic time (from excision to reperfusion) ranged between 83 and 146 minutes (106 ± 22, MW ± SD). During the preparation, hearts were maintained in a cold cardioplegic solution to avoid rewarming.

#### Perfusion system

**Figure 1:**
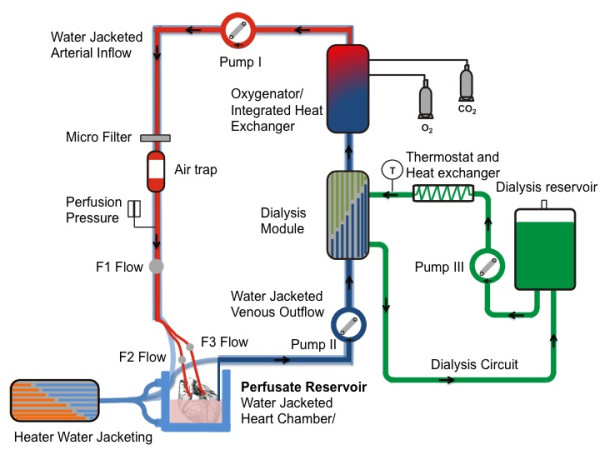
**Schematic view of the perfusion system**. Starting from the perfusate reservoir (38°C) the perfusate is first passed through roller pump 2 into a dialysis module and then through a blood oxygenator with integrated heat exchanger. The oxygenated blood is then pumped into the heart via roller pump 1. Before entering the heart an air trap removes air bubbles from the perfusate. The dialysate (38°C) is pumped through the dialysis module with a centrifugal pump (pump 3, flow of 5 l min -1) to exchange metabolites (contrast agent clearance) between the venous perfusate and the dialysate. The dialysate is re-circulated in a reservoir. T: sensor for dialysate temperature; O2 and CO2: valves for oxygen and carbon dioxide input. The perfusate reservoir and the venous and arterial blood circuits are fully water-jacketed. Temperature is controlled by an external heater.

We performed the experiments in a system for direct coronary perfusion, in which the left and right coronary artery can be perfused independently of each other (Figure 1). The heart was connected to the system with an aortic cannula inserted into the Aorta. The aortic cannula contained catheters to selectively perfuse right and left coronary arteries, to allow precise control of regional myocardial perfusion.

The perfusion system consisted of two separate circuits - the haemoperfusate circuit to supply the heart with nutrients and oxygen and the dialysate circuit for the exchange of metabolites and clearance of contrast agent (Figure 1). To avoid temperature loss the heart was suspended in whole blood in a purpose designed warm water-jacketed heart chamber. Blood temperature and oxygenation were controlled by an oxygenator with an integrated heat exchanger (Dideco D 100, Mirandola, Modena, Italy). The perfusate consisted of autologous whole blood (1.8 litre), diluted with Krebs-Henseleit solution (1 litre, 9.6 g dry mass, Sigma, St. Louis, MO, USA, Krebs-Henseleit buffer K-3753). To adjust the pH, CO_2 _was added if required.

#### Connection to the perfusion system and reperfusion

Following cannulation, hearts were connected to the perfusion apparatus and perfusion of the coronary arteries was started at an initial pressure of 50 mmHg. Approximately 2 minutes after the start of perfusion the pressure was increased to 70 mmHg. In the event of ventricular fibrillation electrical defibrillation was performed. After a stabilisation period of 10 to 15 minutes coronary blood flow (CBF) was adjusted to reach a CPP between 60 and 80 mmHg. All hearts were perfused for 4 hours.

#### Measurements on the isolated hearts

Left ventricular pressure (LVP), right ventricular pressure (RVP), coronary perfusion pressure (CPP) and coronary blood flow (CBF) were assessed every 30 minutes. Arterial blood gas analysis and oximetry were performed every 15 minutes and included partial oxygen (pO_2_) and partial carbon dioxide (pCO_2_) pressure in addition to pH, haemoglobin, sodium, potassium, calcium, chloride, glucose and lactate.

### Phase 2

Within phase 2 hearts were commercially purchased from Harlan Laboratories UK. Hearts were harvested from Large White Cross Landrace pigs weighing between 40 and 60 kg (average weight of 49 ± 5 kg). Due to local requirements the protocol differed as follows: Pigs were sedated with 10 mg/kg i.m. ketamine and 0.3 mg/kg i.m. xylazine. General intravenous anaesthesia was achieved with 1.5 mg/kg i.v. alphaxolone. Coronary arteries were perfused with 20 ml sterile concentrate for cardioplegia infusion (Martindale Pharmaceuticals, Romford, Essex, UK) diluted immediately before use in 1 litre of Ringer's Solution.

#### Modified MR-Compatible perfusion system

**Figure 2:**
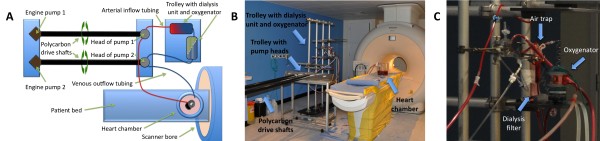
**MR compatible model**. Column A, B and C show the technical setup of the MR compatible perfusion system in detail. **A and B **All electrical parts (control-unit, power-supply, pumps etc.) are placed approximately 6 metres away from the magnet. The pump engines were then connected to the custom made pump heads using polycarbon drive shafts. **C **Detail of the trolley containing dialysis unit and oxygenator.

We re-developed the model to be run in the MR environment. The setup consisted of MR safe and MR compatible parts. All equipment in the scanner was made of MR safe materials [13], i.e. not influencing the MR-homogeneity of the main magnetic field. Electrical and magnetic parts (control-unit, power-supply, pumps, pacer etc.) were made MR compatible by placing them approximately 6 metres away from the magnet outside the 5 Gauss line (Figure 2). The pump engines were connected to the custom-made pump heads using polycarbon drive shafts. The cylindrical chamber for the perfused heart was made of perspex, which has a magnetic susceptibility close to water and therefore these materials are not affecting the B0-homogeneity and thus image quality as described by Schenck [14]. ECG-triggering was performed by means of a small animal MR ECG system (SA Instruments, Stony Brook, NY, USA). Pacing wires were custom made from copper and connected to a clinical pacing system (Biotronik EDP 20, Berlin, Germany). Temperature was monitored by an MR compatible thermocouple. This set-up allowed us to run the same system within the MR scanner as the one we had developed for the non MR environment.

#### Measurement of interference with Image quality of the perfusion system

Designing the perfusion system to be MR-compatible ensures that the equipment does not adversely affect the function of the MR scanner, i.e. no significant image artefacts or noise. Since all equipment inside the MR scanner was made of MR safe material the main source of potential image degradation would be due to radiofrequency (rf)-interference of equipment (e.g. control-unit, power-supply, pumps, pacer etc.) with the MR scanner. In order to test the potential rf-interference, MR images without rf-excitation were acquired with a perfused heart inside the MR scanner. The resulting noise images allowed for detecting any rf-signal within the bandwidth used by the MR image acquisition. In particular, images with a bandwidth of 185 kHz were acquired at three different centre frequencies ensuring an rf sweep across a 550 kHz range around the MR resonance frequency. This range covers the range of frequencies that can be acquired in CMR. To compare this to the background level of noise, the same experiment was also acquired with all equipment switched off as well as without equipment in the room.

#### Cardiovascular magnetic resonance

CMR was carried out on a clinical 3 Tesla MR scanner (Achieva, Philips, Best, The Netherlands) and on a clinical 1.5 Tesla MR scanner (Intera CV, Philips, Best, The Netherlands). For signal reception, a clinical 32-channel coil array was positioned around the heart chamber, which was then placed in the magnet.

CMR data were acquired in short axis and long axis (2-chamber, 3-chamber and 4-chamber view) of the LV. We used a balanced steady state free precession (SSFP) sequence with a repetition time of 3 ms, echo time of 1.5 ms, flip angle 40°, spatial resolution at 2 × 1.6 × 8 mm^3 ^for CINE imaging. For perfusion MR imaging we used a saturation recovery gradient echo pulse sequence accelerated with k-t SENSE with a repetition time of 2.4 ms, echo time of 0.8 ms, flip angle 20°, spatial resolution at 1.3 × 1.3 × 8 mm^3^. A volume of 5 ml of Gadobutrolum at a concentration of 7 mmol/l (Gadovist, Schering, Berlin, Germany) followed by a saline flush of 10 ml was administered either into the common arterial inflow line supplying both coronary arteries or selectively into the RCA or LCA through the aortic cannulae for selective first pass perfusion imaging. Late gadolinium enhancement (LGE) CMR was performed in identical slice orientations using conventional methods with a repetition time of 4.9 ms, echo time of 2.4 ms, flip angle 15°, spatial resolution at 1.7 × 1.7 × 8 mm^3^.

Three hearts at 3 T (n = 3) and three hearts at 1.5 T (n = 3) were perfused for 240 minutes with constant blood flow and oxygenation. In one heart at 3 T (n = 1) blood flow was kept constant for 60 minutes with subsequent occlusion of the RCA for 180 minutes. All hearts underwent CINE imaging, selective first-pass perfusion and LGE imaging.

## Results

### Phase 1

**Figure 3:**
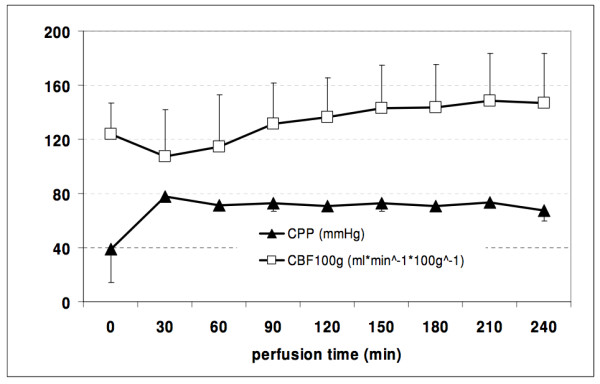
**Average coronary blood flow and perfusion pressure during phase 1**. Coronary Perfusion Pressure (CPP, [mmHg]) and Coronary Blood Flow (CBF [ml/min/100 g]) during 240 minutes of direct coronary perfusion.

Haemoperfusion of six hearts was performed in a direct coronary perfusion mode. Hearts were defibrillated 2-5 times with 30 Joule to reach stable electrical activity with synchronous ventricular contraction. Successful recovery occurred in 6 out of 6 hearts. Heart rate ranged between 60 and 110 beats per minute. Arterial blood gas analysis revealed stable metabolism (Table 1) at a controlled level of coronary perfusion within a near normal physiological range. Normal values of CBF and CPP were maintained throughout the experiments (Figure 3).

**Table 1:** Arterial Blood Gas Analysis and Oxymetry (average and standard deviation for n = 6 pigs).

Perfusion Time [min]	0	15	30	60	90	120	150	180	210	240
**pH**	7,66 ± 0,15	7,55 ± 0,1	7,51 ± 0,14	7,45 ± 0,04	7,42 ± 0,04	7,4 ± 0,03	7,39 ± 0,02	7,38 ± 0,04	7,41 ± 0,03	7,43 ± 0,04

**pO2 **[mmHg]	299 ± 49	265 ± 146	273 ± 86	252 ± 28	215 ± 50	230 ± 40	271 ± 40	282 ± 50	273 ± 55	280 ± 38

**pCO2 **[mmHg]	20 ± 7	26 ± 6	27 ± 5	32 ± 4	34 ± 4	35 ± 3	36 ± 4	36 ± 5	33 ± 5	32 ± 5

										

**tHb **[g/dl]	5,7 ± 0,8	4,9 ± 0,4	5,3 ± 0,6	4,5 ± 0,6	4,5 ± 0,4	4,9 ± 0,8	4,7 ± 0,4	5,2 ± 0,6	4,7 ± 0,6	4,5 ± 0,4

										

**Calcium **[mmol/L]	0,45 ± 0,02	1,16 ± 0,12	1,23 ± 0,1	1,28 ± 0,11	1,28 ± 0,11	1,27 ± 0,11	1,28 ± 0,11	1,28 ± 0,12	1,27 ± 0,11	1,26 ± 0,11

**Potassium **[mmol/L]	5 ± 0,1	5,1 ± 0,1	5,1 ± 0,1	5,1 ± 0,2	5,1 ± 0,2	5,1 ± 0,2	5,2 ± 0,2	5,3 ± 0,2	5,3 ± 0,2	5,3 ± 0,2

**Sodium **[mmol/L]	132 ± 6	131 ± 5	131 ± 4	132 ± 4	132 ± 4	132 ± 4	132 ± 4	132 ± 4	133 ± 4	133 ± 4

**Chloride **[mmol/L]	111 ± 3	110 ± 4	111 ± 5	110 ± 5	110 ± 4	110 ± 4	111 ± 4	110 ± 4	111 ± 4	111 ± 5

										

**Glucose **[mmol/L]	8,7 ± 0,3	8,5 ± 0,1	8,4 ± 0,1	8 ± 0,2	7,8 ± 0,2	7,5 ± 0,1	7,1 ± 0,2	6,7 ± 0,2	6,6 ± 0,2	6,3 ± 0,2

**Lactate **[mmol/L]	0,5 ± 0,2	0,7 ± 0,2	0,9 ± 0,2	1,1 ± 0,2	1,3 ± 0,2	1,5 ± 0,2	1,7 ± 0,2	2 ± 0,3	2,3 ± 0,3	2,6 ± 0,4

### Phase 2

#### Interference with image quality of the perfusion system

The acquired noise images showed no significant rf-interference of the equipment inside the MR room with the MR scanner. In particular, no additional high rf-signals were observed over the complete frequency range of 550 kHz, which would result in strong image artefacts. However, a small increase (15%) of the noise level was found when the electrical equipment inside the MR-room was switched on.

#### Cardiovascular magnetic resonance

**Figure 4:**
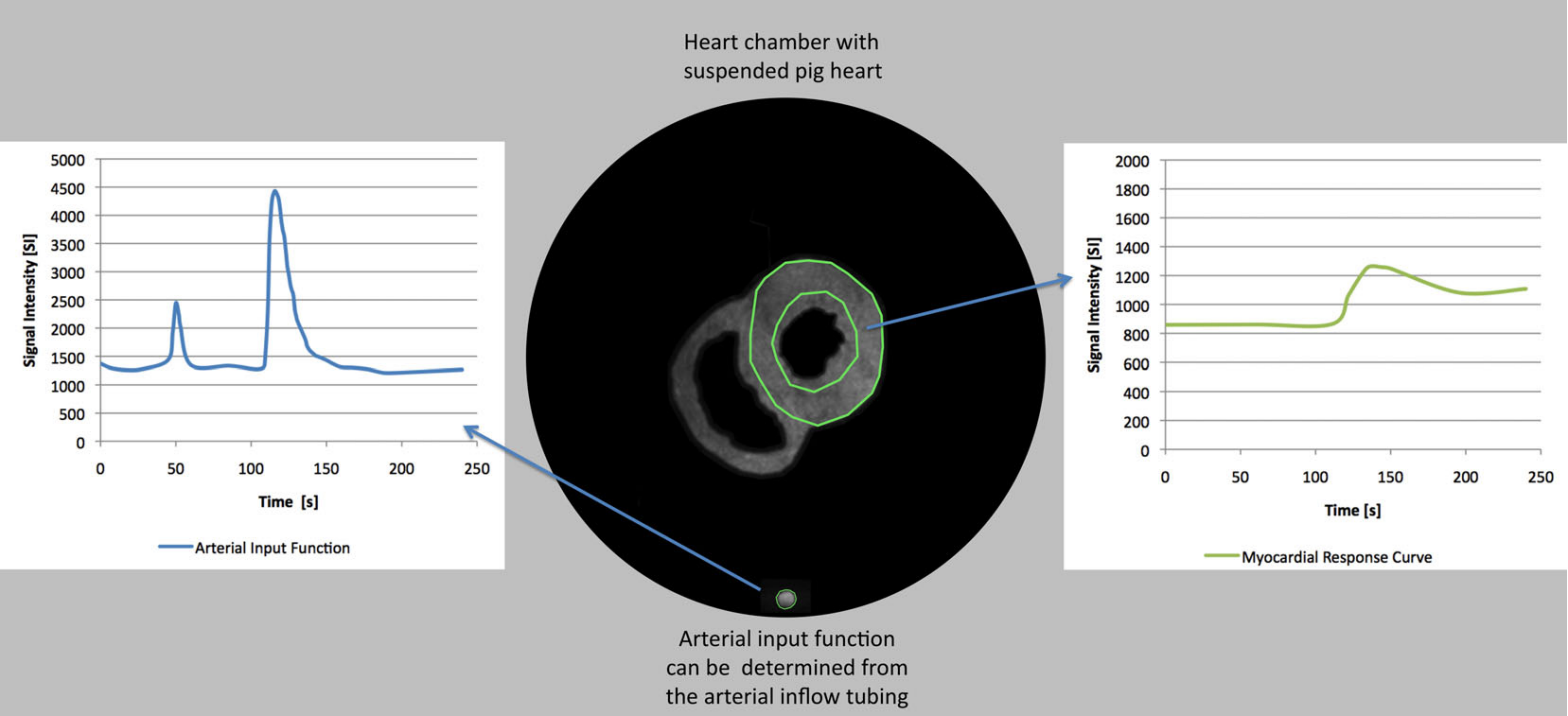
**Determination of signal intensity curves during first pass perfusion in the isolated pig heart**. The figure shows a short axis cut through one isolated pig heart imaged at 1.5 Tesla. The image plane contains a perpendicular cut through the arterial inflow tubing. The arterial input function including prebolus can be obtained from the blood pool during first pass of gadolinium. The myocardial response curve can be obtained shortly afterwards during wash-in of contrast media into the myocardium. The images have been segmented to improve visibility.

**Figure 5:**
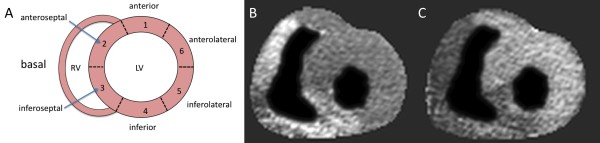
**Selective perfusion imaging of the right coronary artery (RCA) and left coronary artery (LCA) perfusion territories in the isolated pig heart**. Figure 5 show two short axis slices during first pass perfusion of gadolinium imaged at 3 Tesla after selective injection into the right and left coronary artery, respectively. **A **Anatomic reference plane (basal slice). **B **k-t SENSE selective RCA first pass perfusion. **C **k-t SENSE selective LCA first pass perfusion. The images have been segmented to improve visibility.

**Figure 6:**
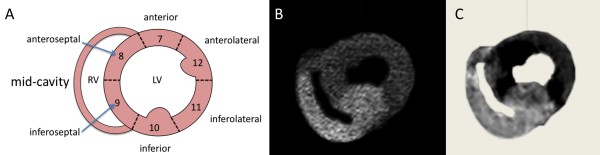
**Perfusion territory and Size of Infarction**. The perfusion territory of the RCA was mapped in the 3 Tesla scanner by first pass perfusion with direct injection of the contrast agent into the RCA (central image). The perfusion territory was 96 g (36% of left ventricular myocardial mass). Then the RCA was occluded for 180 minutes. Late gadolinium enhancement (LGE, image on the right) resulted in 95 g myocardial infarction (36% of LV mass). The close agreement of LGE and selective RCA perfusion highlights the almost complete absence of collaterals in pigs. **A **Anatomic reference plane (midmyocardial slice). **B **k-t SENSE selective RCA first pass perfusion. **C **LGE image after 180 minutes of RCA occlusion. The images have been segmented to improve visibility.

Hearts were defibrillated with 30 Joule 3 times on average. We performed CINE imaging, first pass perfusion imaging and LGE imaging. The image quality was comparable to clinical scanning (Figure 4, 5 and 6).

Signal intensity curves during first pass perfusion imaging were obtained in a view perpendicular to the heart chamber. The image plane contained a perpendicular cut through the arterial inflow tubing (arterial input function) and a short axis view of the myocardium (myocardial response curve) (Figure 4).

By selective injection of gadolinium contrast agent into RCA and LCA the spatial extent of the respective territories was determined (Figure 5).

In one heart at 3 T blood flow was kept constant for the first 60 minutes. After standard assessment of ventricular function the perfusion territory of the RCA was mapped by first pass perfusion (Figure 6B). The RCA was subsequently occluded for 180 minutes. We found good agreement between the left ventricular myocardium in the RCA territory identified by first pass perfusion prior to RCA occlusion and the left ventricular territory identified by LGE (Figure 6C) imaged 180 minutes after RCA occlusion. The RCA territory measured using the short axis RCA first pass perfusion stack was 96 g representing 37% of the total (258 g) left ventricular myocardial mass. The RCA territory measured using LGE imaging which represents infarction was 95 g or 36% of the total LV mass (Figure 6).

## Discussion

The data presented here demonstrate the potential of an isolated, blood-perfused pig heart preparation as an experimental tool to validate newly developed CMR techniques and assess numerous physiological parameters.

Our study demonstrates that 1.) stable near physiological conditions are obtained during normal perfusion and oxygenation of the heart, 2.) the model can be safely run in an MR scanner, 3.) interventions such as down-regulation of flow or occlusion of a coronary artery can be easily performed and 4.) imaging of the beating, isolated, blood-perfused pig heart system is feasible in a clinical system using imaging sequences identical to those used for patient examinations.

Pigs have been widely used in cardiovascular research. Cardiac energetics have been studied in stunned, hibernating and hypertrophied myocardium [15-18]. Graded ischaemia [19], ischaemic preconditioning [20] and the coronary microcirculation [21] have all been studied in different porcine models. *In vivo *imaging in pig models has previously been used to study coronary blood-flow using coloured microspheres [22], microvascular function using computer tomography (CT) [23], and to validate novel perfusion tracers for positron emission tomography (PET) [24]. Other applications include validation of MR-guided real time coronary catheterization or percutaneous transluminal balloon angioplasty in aortic coarctation [25,26].

Advances in MR such as novel contrast agents [27], T2 weighted sequences for the detection of oedematous tissue [28] or quantitative assessment of myocardial perfusion were initially validated in pigs [29]. Schmitt et al. demonstrated correlation between experimentally reduced coronary perfusion measured with quantitative CMR and fluorescently labelled microspheres [30]. Recently Christian and co-workers elegantly used this animal model to compare CMR quantitative myocardial perfusion imaging at 1.5 and 3 Tesla [31].

The isolated pig heart model is a useful tool. Whilst it is not as physiological as intact animals it offers much greater control and reproducibility. It can be run in a coronary perfusion mode or in a working heart mode and can either be perfused with crystalloid solution or whole blood.

Previous work has been performed in hearts from the abattoir [10,32] or in surgically explanted hearts [33]. The pathophysiology of cardiac arrhythmias and electrical conduction of endocardial pacing were studied in coronary perfused pig hearts [33,34]. This perfusion mode was also used to study right and left ventricular performance in neonatal hearts [35] and to compare different techniques of donor heart preservation [11].

Furthermore preclinical high-field Rubidium-87 MR spectroscopy imaging at 7 Tesla has been used to study ischaemia and infarction in coronary blood-perfused pig hearts [36].

Modersohn and co-workers studied transmyocardial laser revascularisation in a 2-chamber (left atrium and left ventricle) working heart [37] whilst Chinchoy et al. studied cardiac physiology in a crystalloid solution perfused 4-chamber working heart [38]. Intracardiac digital imaging of valvular motion [39] and fluorimetric evaluation of function [12] were also investigated under working conditions.

Finally isolated non-beating porcine hearts have been previously investigated by MR myocardial perfusion imaging to compare kinetics of different contrast agents and to investigate the microcirculation of transplant hearts [40,41].

The present study to the best of our knowledge is the first to demonstrate the feasibility of perfusing a beating heart system in a clinical MR scanner. Our current model has several important advantages over other experimental designs, but also some limitations, which need to be considered. The main advantage is the model's precision allowing accurate control of regional and global perfusion and oxygenation, as well as controlled nutrient delivery. Whole heart or regional ischaemia can easily be induced and adjusting perfusion can control the severity of ischaemia. Pig hearts are well suited for studies of myocardial blood flow as they have virtually no anastamoses between adjacent coronary perfusion beds (Figure 6) [8]. This anatomical homogeneity means that studies are much more reliable and reproducible. The model is also free from external influences such as neuro-humoral activation- the effects of which are difficult to quantify. In addition in contrast to in vivo preparations the isolated heart preparation allows experiments to be continued after fatal events (e.g. infarction induced cardiac arrest or arrhythmias), which can frequently terminate an in vivo experiment.

Despite making the system MR compatible we were able to keep the short tubing and the small volume of the circuit. This is important as it allows the use of a mixture of autologous blood and crystalloid solution with near normal haematocrit and haemoglobin without the need for donor or support animals. Perfusion with blood leads to more stable function and less oedema compared to crystalloid solution perfusion [8].

The main disadvantages of this model are the less physiological environment compared to in vivo experiments, the ischaemic period after heart removal and the high associated costs.

## Limitations

For studies of myocardial perfusion rapid recirculation of contrast is prevented in the current model. This is an oversimplification of in vivo conditions but allows for repeated perfusion scans. In addition contrast arrival time was longer compared with in vivo as a consequence of distribution and dilution in the inflow-tubing. Whether this influences quantitative perfusion analysis with CMR needs to be investigated.

As this was a feasibility study we did not perform metabolic assessment of the 6 hearts we perfused in the magnets. Therefore stable physiological function was assumed on the basis of the previously performed validation experiments. Future trials aiming to answer specific questions need to include metabolic measurements relevant to the nature of the hypothesis and to assure stable, robust and reproducible conditions for the specific scientific question.

We performed a proof of concept study to demonstrate the feasibility of the model in the MR environment and thus we did not perform systematic statistical analysis.

## Conclusions

The current work demonstrates for the first time the feasibility of operating and imaging an isolated beating porcine heart preparation in a clinical MR scanner. The technical design of this isolated pig heart model allows ex vivo simulation and imaging of cardiac function. This novel system provides excellent control of physiological parameters and allows validation of novel techniques against gold standards and easy translation of the methods to patients using identical equipment and imaging sequences.

## Competing interests

Tobias Schaeffter served as a Consultant to Philips Healthcare. Eike Nagel received minor consultancy fees from GE and Philips Healthcare and minor speaker honoraria from GE, Philips Healthcare, and Bayer Schering Pharma. The other authors declare that they have no competing interests'.

## Authors' contributions

AS carried out the perfusion studies, designed and implemented the MR compatible model and drafted the manuscript. IG carried out the perfusion studies and substantially contributed to the design of the MR compatible model. AC carried out the perfusion studies, designed and implemented the MR compatible model. RS participated in the design of the MR compatible model and helped with the perfusion studies. MI helped with the perfusion studies, and participated in the study design. GH and NN helped with the perfusion studies. GM and DP participated in the study design and helped to draft the manuscript. TS participated in the sequence alignment and helped to draft the manuscript. EN designed and implemented the MR compatible model and drafted the manuscript. All authors read and approved the final manuscript.
